# Genome-wide identification of bHLH transcription factors: Discovery of a candidate regulator related to flavonoid biosynthesis in *Erigeron breviscapus*

**DOI:** 10.3389/fpls.2022.977649

**Published:** 2022-09-14

**Authors:** Qingqing Gao, Wanling Song, Xia Li, Chunfan Xiang, Geng Chen, Guisheng Xiang, Xiangyu Liu, Guanghui Zhang, Xiaoning Li, Shengchao Yang, Chenxi Zhai, Yan Zhao

**Affiliations:** ^1^Key Laboratory of Medicinal Plant Biology of Yunnan Province, National and Local Joint Engineering Research Center on Germplasms Innovation and Utilization of Chinese Medicinal Materials in Southwest China, Yunnan Agricultural University, Kunming, China; ^2^College of Agronomy and Biotechnology, Yunnan Agricultural University, Kunming, China; ^3^Sibley School of Mechanical and Aerospace Engineering, Cornell University, Ithaca, NY, United States

**Keywords:** *EbbHLH80*, flavonoids, bHLH transcription factors, anthocyanins, *Erigeron breviscapus*

## Abstract

*Erigeron breviscapus* is a Compositae plant, and its rich flavonoids have shown strong preventative and curative effects in the treatment of cardio- and cerebrovascular diseases. *bHLH* genes play a crucial role in plant growth and development. There are 116 *EbbHLH* genes in *E. breviscapus*, and each gene has been named based on its chromosome location. Our phylogenetic analysis divided these genes into 18 subfamilies. To further investigate its function, *EbbHLH80* was isolated from *E. breviscapus* leaves. Next, transcriptomic and metabolomic analyses of tobacco leaves were performed. Among 421 differentially accumulated compounds, 98 flavonoids were identified. In addition, differentially expressed genes were identified using RNA-seq, and further analysis suggested that *EbbHLH80*-OE could not only regulate the expression of some structural genes in the flavonoid biosynthesis pathway to achieve flavonoid accumulation but also be involved in the regulation of a series of downstream pathways, such as stress response, ABA and ethylene signal transduction, to affect plant growth and development. The results of our analysis provide new insights into the function of *EbbHLH80* and lay the foundation for future functional studies on *E. breviscapus*.

## Introduction

As one of the largest transcription factor families, basic helix-loop-helix (bHLH) proteins are one of the most abundant. In plants, fungi, and animals, these transcription factors (TFs) are widely distributed (Riechmann and Muyldermans, 1999; Carretero-Paulet et al., 2010). A bHLH TF is composed of two connected subregions, a basic region located directly following the helix-loop-helix (HLH) domain and a dimerizing region consisting of 40–50 amino acid residues (Atchley et al., 1999; Carretero-Paulet et al., 2010). *bHLH*s play an important role in regulating flavonoid biosynthesis and ROS (reactive oxygen species) homeostasis under stress. There has been an increase in the number of bHLH transcription factors found in plant species that regulate flavonoid biosynthesis pathways, and a key function of these transcription factors is to regulate anthocyanin biosynthesis (Jin et al., 2018).

Previous studies have identified several enzyme-coding structural genes involved in phenylpropanoid, flavonoid and anthocyanin biosynthesis (Chen et al., 2019; Sun et al., 2020). It has been demonstrated that the *bHLH* transcription factor *TRANSPARENT TESTA8* (*ATT8*; AT4G09820) regulates the flavonoid biosynthesis pathway via the transcription of key enzymes BAN (Banyuls) and DFR (dihydroflavonol 4-reductase) (Nesi et al., 2000; Baudry et al., 2004). *TT8* is an important transcription factor involved in secondary metabolism as well as the response to stress (Zhai et al., 2020). *TT8* loss-of-function lines show altered chromatographic profiles for aglycones of kaempferol and quercetin as well as glycosylated forms (Pelletier et al., 1999; Narasimhan et al., 2003). This complex of R2R3-MYB, bHLH, and WD40 repeat (MBW) proteins directly binds to promoters of flavonoid biosynthesis genes (Dubos et al., 2010; Xu et al., 2014). Previous studies have shown that the flavonoid pathway is regulated in plants by different members of the *bHLH* family, such as *CmbHLH2*, which significantly activates *CmDFR* transcription and triggers anthocyanin accumulation while coexpressed with *CmMYB6* in Chrysanthemums (*Chrysanthemum morifolium* Ramat.) (Xiang et al., 2015). In the inner pericarp of *Actinidia chinensis* cv. Hongyang, coexpression of *AcMYB123* and *AcbHLH42* activates *AcF3GT1* and *AcANS* or their homologous gene expression, promoting anthocyanin accumulation (Wang et al., 2019). Moreover, *AabHLH1* interacts with *AaMYB3* to regulate the accumulation of procyanidin (Li et al., 2019). *DhMYB2* was identified in *Dendrobium* hybrids, interacting with *DhbHLH1* to regulate anthocyanin production (Li et al., 2017). Additionally, *PPLS1*, a *bHLH* transcription factor, interacts with *SiMYB85* to regulate anthocyanin biosynthesis in *Setaria italica* (Bai et al., 2020). A series of transporters facilitate the transportation of flavonoids to vacuoles after biosynthesis, including glutathione S-transferases (GSTs), multidrug resistance-associated proteins (MRPs), and multidrug and toxic compound extrusion (MATE) proteins (Marrs et al., 1995; Mueller et al., 2000; Zhao and Dixon, 2009).

In the Compositae family, *Erigeron breviscapus* (*E. breviscapus*) is an important medicinal plant and is rich in flavonoids in its leaves (Su et al., 2001; Chu et al., 2005). Scutellarin is one of the major active components and is widely used as a prescription drug. *E. breviscapus* was the first medical plant to undergo an older version genome assembly to accomplish the process from genome sequencing to metabolite biosynthesis, and genome assembly and engineering yeast were used to produce breviscapine (Liu et al., 2018; He et al., 2021). In addition, extensive studies on *bHLH* gene family members in horticultural and crop plants have increased our understanding of their functions and transcriptional regulatory mechanisms. Moreover, extensive research has been conducted on *bHLH* genes in horticultural plants, learning more about their regulatory mechanisms. However, it is unclear whether *bHLH* genes are associated with regulating flavonoid biosynthesis in *E. breviscapus*. A recent publication of the genome sequence of *E. breviscapus* provided a way to identify the *bHLH* gene family (He et al., 2021).

Therefore, whether *EbbHLH80* has a similar function as the *AtTT8* regulator in regulating the flavonoid pathway, is worthy of further investigation. In previous studies, heterologous overexpression of *bHLH*s has been shown to regulate the synthesis of flavonoids. Many *bHLH*s have been found to play a positive role in regulating flavonoid synthesis. The grape *VvbHLH1* gene is heterologously overexpressed and increases enzyme activity related to flavonoid synthesis (Wang et al., 2016b). *VvbHLH003* and *VvbHLH007* were also found to be related to flavonoid synthesis (Wang et al., 2018). Similarly, *Arabidopsis PAP2*, a MYB transcription factor, is heterologously expressed and increases anthocyanin contents in tomatoes (Li et al., 2018), while the liverwort (*Plagiochasma appendiculatum*) *PabHLH1* is heterogeneously overexpressed in *Arabidopsis* and induces both flavonoid and anthocyanin synthesis by upregulating structural genes involved in flavonoid synthesis (Zhao et al., 2019). Furthermore, apple *MdMYC2* is ectopically overexpressed and significantly upregulates the expression of structural genes in transgenic *Arabidopsis* (An et al., 2016). In previous studies, *TsMYC2* from triticale (*Triticum* × *Secale*) and *TaMYC1* from wheat (*Triticum aestivum*) were shown to participate in accumulating anthocyanin and regulate the grain properties associated with the blue aleurone trait (Zong et al., 2019).

Here, we transferred *EbbHLH80* into tobacco and observed ectopic flavonoid accumulation. By further integrating metabolome and transcriptome analysis *EbbHLH80-OE* and Yunyan87 (WT), we identified key metabolites and genes (structural genes or TFs) in leaves to pinpoint key genes controlling metabolite composition in *EbbHLH80-OE* and Yunyan87. This enabled us to investigate the coordinated regulation of metabolite components and other biological processes directly coregulated with metabolites. Finally, *EbbHLH80* was suggested to be an integrator of secondary metabolism TFs that can regulate flavonoid biosynthesis.

## Materials and methods

### Plant materials

*Erigeron breviscapus* seeds were obtained from Longjin Biotech Co., Ltd. (Xuanwei, Yunnan, China). *Nicotiana tabacum* (Yunyan 87) seeds were obtained from Yunnan Agricultural University (Kunming, Yunnan, China). The flowers, leaves, roots and stems of *E. breviscapus* that grew for approximately 8-week-old were collected to quantify *EbbHLH80* in the different tissues. Three biological replicates were collected for each sample. The roots, stems, leaves and flowers of *E. breviscapus* and leaves of tobacco were frozen with liquid nitrogen and then stored at −80°C.

### Identification and sequence analysis of the *EbbHLH* gene family

The Hidden Markov Model (HMM) profile of the bHLH DNA-binding domain (PF00010) downloaded from the Pfam database^1^ was used for the identification of *bHLH* genes in the *E. breviscapus* genome (He et al., 2021) using the simple HMM search program TBtools (Chen et al., 2020). The ExPASy proteomics server was used to determine the molecular weights of EbbHLH proteins and their isoelectric points.^2^ Subcellular localization was predicted by Plant-mPLoc.^3^

### Gene structure and conserved motif analysis of *EbbHLH* gene family

The NCBI Conserved Domain Search^4^ was used to test for the presence of the bHLH domain. TBtools software was used to visualize NCBI CDD domain patterns (Chen et al., 2020). To analyze the motifs of EbbHLH proteins, the online Multiple Expectation Maximization for Motif Elicitation (MEME)^5^ was selected and used (Bailey et al., 2006). Furthermore, EbbHLH protein sequences were identified using the MEME program of TBtools. The following parameters were optimized for MEME: AnyNumberOfOccurPerSeq; 15 motifs to be found; and 6–60 residues for each motif. TBtools was also used to visualize MEME results (Chen et al., 2020).

### Chromosomal distribution and phylogenetic analysis *EbbHLH* gene family

According to the positions assigned in genome annotations of *E. breviscapus*, *EbbHLH* genes were located on its chromosomes using TBtools software (Chen et al., 2020). For the phylogenetic analysis, the multiple sequence alignments of the bHLH proteins from *E. breviscapu*s and *Arabidopsis* were aligned by the ClustalW program and adjusted manually. The phylogenetic tree was constructed by the neighbor-joining method in MEGA 7.0 with 1,000 bootstrap replications (Kumar et al., 2016). The same method was adopted to construct phylogenetic trees of bHLH proteins from *E. breviscapu*s. The phylogenetic trees of all EbbHLH and 120 AtbHLH were constructed using the same method described above, and the AtbHLH sequences were downloaded from TAIR (The *Arabidopsis* Information Resource).^6^ According to the phylogenetic tree, certain bHLH proteins were predicted to have certain biological functions.

### Stable transgenic tobacco

The CDS of *EbbHLH80* was cloned from leaves of 8-week-old *E. breviscapus.* The PC1300-35S-*EbbHLH80* construct was generated by subcloning the target fragments between the *BamH*I and *Xba*I sites of the PC1300-35S vector using the primers listed in Supplementary Table 1. Then, the plasmids were transformed into *Agrobacterium tumefaciens* GV3101, which was then used in tobacco cultivar Yunyan87 (*Nicotiana tabacum*) transformation with the leaf disc methods described by Huang et al. (2016). Regeneration shoots and healthy resistant shoots were selected on the selective shooting medium and rooting medium, both containing 50 mg ⋅ L^–1^ kanamycin and 250 mg ⋅ L^–1^ carbenicillin. Well-developed rooted plants were transferred to soil and then grown in a growth room at 25 ± 2°C with 65–70% relative humidity.

The leaves samples were obtained from 7-week-old Yunyan87 (wild-type, WT) and homozygous T2 lines of transgenic tobacco plants (*EbbHLH80-OE*), were harvested for transcriptome and metabolome sequencing. Liquid nitrogen was used to freeze all samples immediately, and then they were stored at -80°C. Three biological replicates were used in this study, each comprising leaves from six individual plants.

### Measurement of total flavonoids content

To quantify the total flavonoids content, the leaves were harvested from six different 7-week-old tobacco plants. Total flavonoids were extracted and quantified using the method described by Wang et al. (2016a). A spectrophotometer (UV-1800, Shimadzu) was used to measure the absorbance of the samples at 535 nm. Methanol containing 1% HCl was used as a blank control. Content of flavonoids was calculated by Zhao et al. (2022), and data was expressed as mg/g FW. For each sample, at least three biological replicates were performed.

### Metabolic analysis

Wild-type (WT) and *EbbHLH80*-OE metabolite profiling was performed using a widely targeted metabolome method with three biological replicates in each group (Chen et al., 2013). Quality control (QC) analysis was conducted before the data analysis. Analyst 1.6.1 was used to filter data, detect peaks, align, and perform all other calculations. A variable importance in projection (VIP) score of the (O)PLS model was applied to rank the most clearly distinguished metabolites between groups. Metabolites with significant differences in content were defined as having a variable importance in the project (VIP) ≥ 1 and *T*-test *P* < 0.05.

### Transcriptome analysis

The leaves were obtained from 7-week-old WT and *EbbHLH80-OE* tobacco for transcriptome analysis with three biological replicates in each group. The total RNA was extracted for reverse-transcribing into cDNA to construct a cDNA library. To perform the quality control, the raw data was controlled by fastp. RNA purity and integrity were determined with 1% agarose gel, Nanophotometer spectrophotometer, and Agilent 2100 Bioanalyzer. These data were subsequently used to analyze base composition and mass distribution to confirm the quality of this set of data. HISAT2 software was used for alignment analysis with the reference genome, the transcripts of the new genes were assembled with Stringtie, the gene expression level was analyzed with FPKM, the significantly differentially accumulated genes were screened according to FDR < 0.05 and log2FC > |1|, Gene Ontology (GO) functional and Kyoto Encyclopedia of Genes and Genomes (KEGG) pathways were enriched with TBtools software (Chen et al., 2020) and the KEGG database.

### Validation of gene expression using quantitative real-time PCR

The RNA extraction kit (Magen, China) was used to extract total RNA from the samples. RNA was reverse-transcribed into cDNA using a reverse transcription kit (Takara, China). In accordance with qPCR SYBR Green Master Mix, a 20 μL reaction system was constructed (Vazyme, China). PCR was performed on a QuantStudio 5 (ABI) instrument (Thermo Fisher Scientific, Singapore) with the primers listed in Supplementary Table 2. The *NtActin* gene was used as an internal control, thus obtaining true differences in gene of interest specific expression. The quantitative real-time PCR (RT-qPCR) cycling conditions were as follows: 95°C for 30 s, 40 cycles of 95°C for 10 s and 60°C for 30 s, followed by 95°C for 15 s, 60°C for 60 s, and 95°C for 15 s. After amplification, the melting curves were used to verify the PCR products. Gene expression levels were analyzed by the 2^–ΔΔCT^ method (Livak and Schmittgen, 2001).

### Statistical analysis

Statistical analysis was conducted with one-way analysis of variance. Values of *P* < 0.05 or *P* < 0.01 were considered to be statistically significant. Double and single asterisks indicate significant differences between comparing samples at 0.01 and 0.05, respectively.

## Results

### Identification of bHLH in *Erigeron breviscapus* genomes

A total of 159 bHLH TFs were identified. After removing redundant proteins by BLASTP with NCBI Batch CD-Search tools, 116 bHLH TFs remained (Supplementary Table 3). All *bHLHs* were named *EbbHLH*1–*EbbHLH*116 according to their chromosomal distribution, with details including the renaming, physical position, isoelectric point (pI), molecular weight and length of the coding protein (Supplementary Table 4).

In addition, analysis of EbbHLH physicochemical properties showed that the shortest EbbHLH is EbbHLH050, with 89 amino acids, and the longest is EbbHLH113, with 870 amino acids. The molecular weight (MW) of the proteins ranged from 10.0 (EbbHLH050) to 100.1 kDa (EbbHLH113), and their isoelectric points (PI) ranged from 4.8 (EbbHLH039) to 10.11 (EbbHLH014). Subcellular localization predicted that 115 EbbHLH proteins were located in the nucleus. One EbbHLH protein was found in the mitochondrion (Supplementary Table 5). The large-scale gene expression required for the biogenesis and functional operation of mitochondria is controlled by a related network of TFs and regulators that bind to nuclear DNA.

### Chromosomal localization of *EbbHLHs* in the *Erigeron breviscapus* genome

**FIGURE 1:**
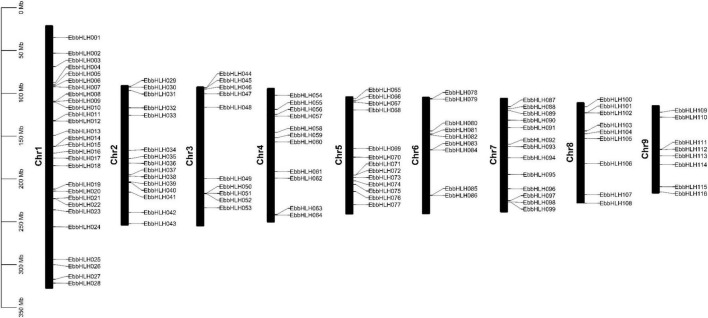
Chromosome distribution of *EbbHLHs* gene. The chromosome number is shown in the middle of each chromosome.

The chromosomal location analyses revealed that the 116 *EbbHLH* genes were randomly distributed on 9 chromosomes (Figure 1). The results showed that 28 *EbbHLH* genes were found on chromosome 1, followed by 15 *EbbHLH* genes located on chromosome 2. In addition, 8 *EbbHLH* genes were located on chromosome 9. There were no significant correlations between chromosome length and the number of *EbbHLH* genes.

### Gene structure and motif composition of *EbbHLHs* in *Erigeron breviscapus*

**FIGURE 2:**
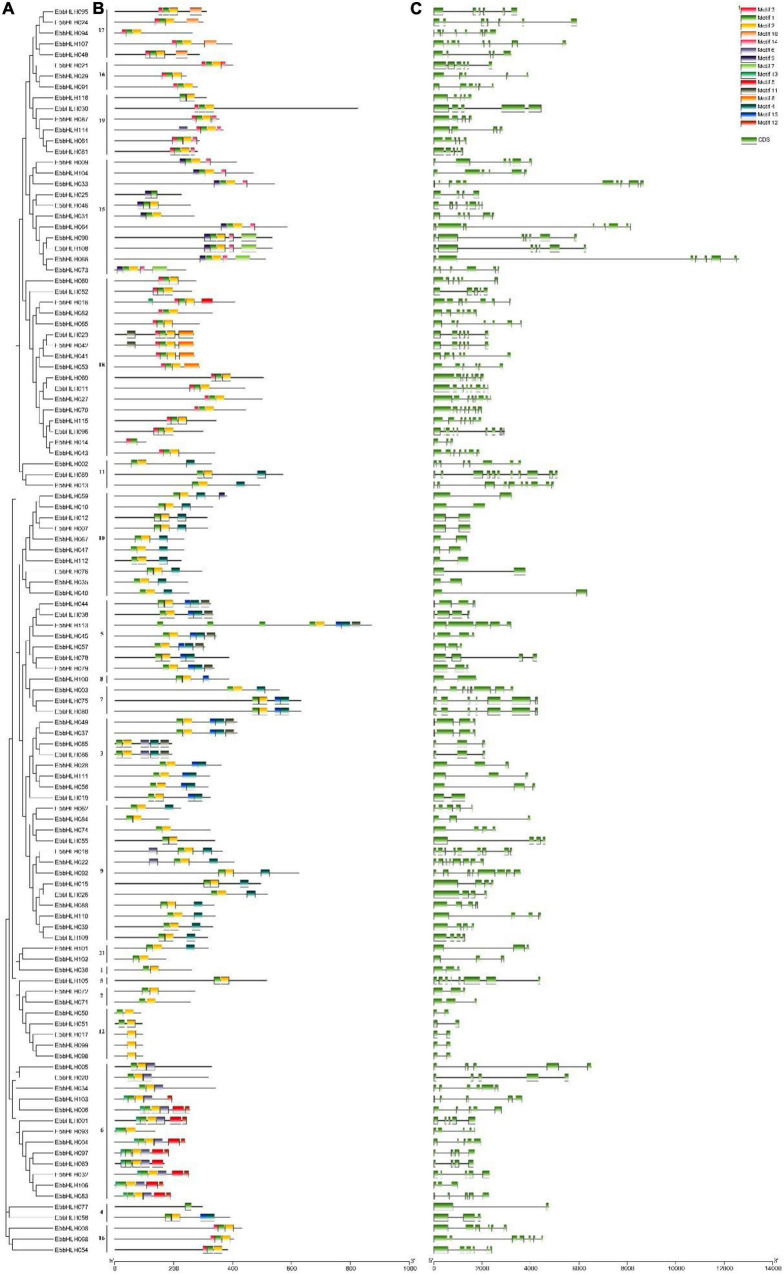
Phylogenetic analysis, gene structure and motif distribution analysis of the *bHLH* gene family of *E. breviscapus*. **(A)** Phylogenetic trees were constructed by the NJ method, repeated 1,000 times at each node. **(B)** Fifteen amino acid motif in the EbbHLH protein is indicated by a colored box. **(C)** Exons and introns are indicated by green rectangles and gray lines, respectively.

Further analysis of the gene structure and the conserved domains in *EbbHLHs* and their exon and intron structures were obtained by comparing the corresponding genomic files (Figure 2C). A comparison of the number and position of the exons and introns revealed that the 116 *EbbHLH* genes have different numbers of exons, varying from 2 to 11. In addition, 18 genes (16%) contained 2 exons, and the remaining genes had 3 or more exons. The largest proportion of *EbbHLH* genes (n = 21) had 3 introns. Furthermore, members of subfamilies 8, 10, and 12 contained only 1 intron, and the remaining protein sequences had more than 2 introns. *EbbHLH089* had the largest number of 10 introns. Both *EbbHLH013* and *EbbHLH033* had 9 introns. In general, all the members of different subfamilies had similar numbers of CDS-intron junctions.

Then, 15 conserved motifs were identified in *E. breviscapus* bHLH proteins (Figure 2B). Eighty-six percent of the EbbHLH protein sequences contained both motifs 1 and 2. Each bHLH protein had a conserved motif position. In addition to the highly conserved bHLH domain, EbbHLH members within the same subgroups usually shared similar motif compositions, while high variance was observed among different subgroups, indicating conserved protein architectures within a specific subgroup. Moreover, some subgroup-specific motifs were detected, which might be required for subgroup-specific functions. For example, motifs 1, 2, 4, and 15 were found in all members of subgroup 5, while motifs 1, 2, and 4 were found in all members of subgroup 9. However, some motifs were only found in specific subgroups. For example, motif 12 was only found in subgroup 6, and motif 10 was unique to subgroup 17, whereas motif 9 was detected in subgroups 10 and 15.

### Phylogenetic analysis of *EbbHLH* genes

**FIGURE 3:**
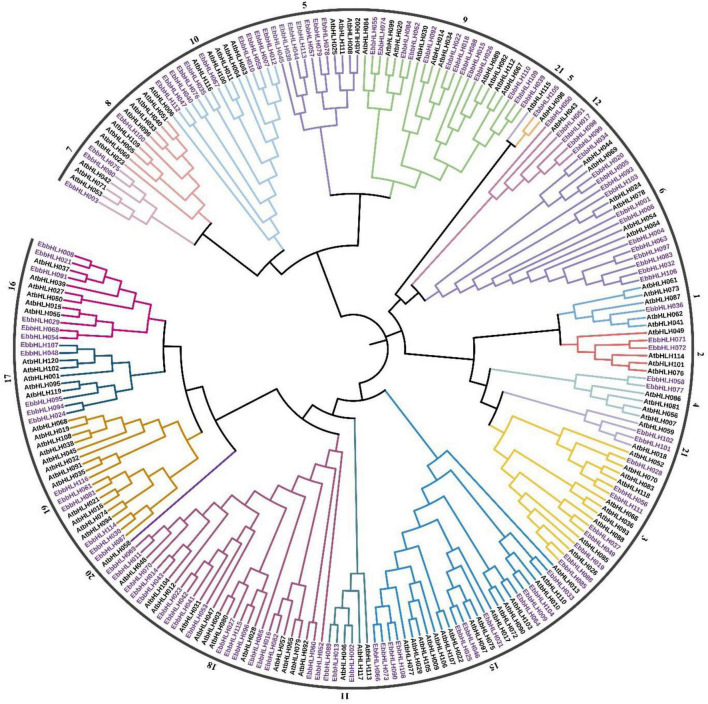
Phylogenetic tree between *E. breviscapus* and *Arabidopsis thaliana*. The phylogenetic tree was obtained using the NJ method in MEGA7. The tree shows 21 phylogenetic subfamilies, each subfamily represented by different colors. bHLH protein from *Arabidopsis* is labeled with the prefix “At.”

To investigate the evolutionary relationships of *EbbHLH* genes in *E. breviscapus*, a neighbor-joining method was constructed with amino acid sequences of 116 EbbHLHs from *E. breviscapus* and 120 AtbHLHs from *Arabidopsis* (Figure 3). In phylogenetic analysis, EbbHLH proteins were divided into 18 subfamilies (Figure 2A), and their regulatory roles were predicted based on AtbHLH classification. Of these, 17 EbbHLHs were assigned to subfamily 18; 13 EbbHLHs were clustered into subfamily 6 and subfamily 9. However, only 1 EbbHLH was assigned to subfamily 1, subfamily 5, and subfamily 8. Additionally, none of the EbbHLHs were assigned to subfamilies 13, 14, or 20. Moreover, the majority of the EbbHLH gene members of subfamily 7 might be regulatory genes of flavonoid biosynthesis. Of all 116 *EbbHLH* genes, *EbbHLH80* was most similar to *AtbHLH042*, which is known to be associated with flavonoid biosynthesis. Due to the high homology with *AtbHLH042* (*AtTT8*), it is worth investigating whether *EbbHLH80* serves a similar function.

### Ectopic expression of *EbbHLH80* promotes flavonoid accumulation in tobacco

**FIGURE 4:**
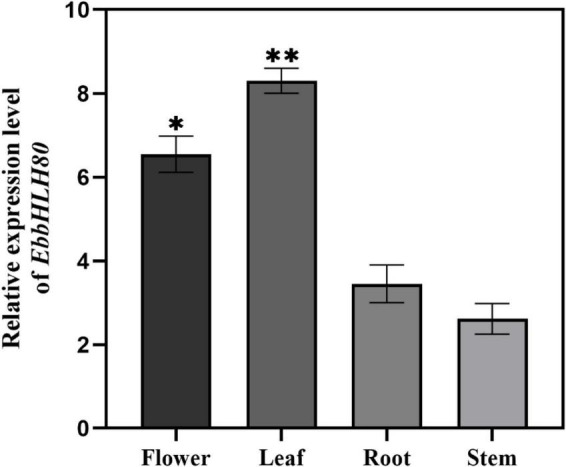
Relative expression of *EbbHLH80* in the different tissues of *E. breviscapus*. Data represent mean ± SD of three biological replicates and the one-way analysis of variance was used for statistical analyses (*P < 0.05; **P < 0.01).

**FIGURE 5:**
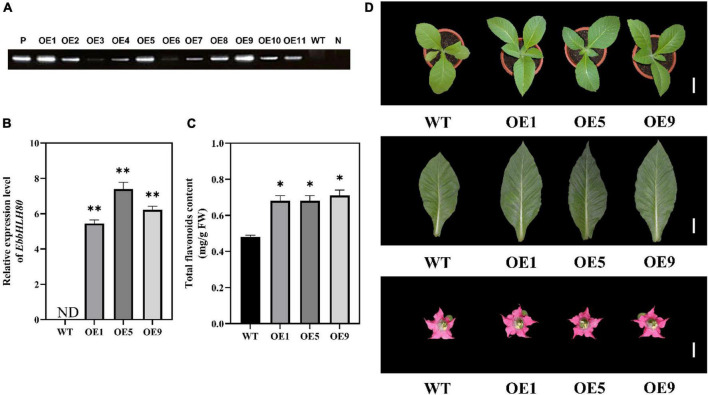
Characterization of transgenic tobacco lines overexpression *EbbHLH80*. **(A)** PCR amplification of *EbbHLH80* in transgenic lines (OE1-11) and WT plants. P, positive control. N, negative control (double-distilled water, ddH_2_O). **(B)** Relative expression level of *EbbHLH80* in WT and *EbbHLH80*–OE (OE1, OE5, and OE9) tobacco transgenic plants. **(C)** Total Flavonoids content in leaves from 7-week-old plants from WT as well as *EbbHLH80*–OE (OE1, OE5, and OE9) tobacco transgenic plants. **(D)** The phenotypes of OE1, OE5, and OE9 tobacco transgenic plants and WT. Bar = 1 cm. Data represent mean ± SD of three biological replicates and the one-way analysis of variance was used for statistical analysis (*P < 0.05 and **P < 0.01).

In our previous study, *EbPAL*, *EbCHS*, and *EbCHI* genes exhibited high expression levels in leaves, while the main component scutellarin, is rich in the leaves, followed by flowers, stems, and roots (Zhao et al., 2022). To analyze the expression of *EbbHLH80*, the results showed that the highest amount of *EbbHLH80* was found in the leaves of *E. breviscapus* (Figure 4), which was used in subsequent experiments. To investigate the possible function of *EbbHLH80* in flavonoid biosynthesis, the complete ORF of *EbbHLH80* (1,896 bp) was isolated and obtained, and then the *35S:EbbHLH80* construct ectopically expressed *EbbHLH80* in tobacco (Figure 5A). Several independent *35S*:*EbbHLH80* transgenic tobacco lines were screened, and three transgenic lines (OE1, 5, and 9) showing higher *EbbHLH80* expression levels than other lines were selected for subsequent experiments (Figures 5B,D). The transcription levels of the *EbbHLH80* genes in the WT and OE lines were analyzed. Compared with WT, the expression levels of *EbbHLH80* in OE lines were significantly increased. Next, we quantified the level of total flavonoids in transgenic and WT plants. The total flavonoid content of OE1, OE5, and OE9 transgenic lines was significantly higher by 1. 41-, 1. 49-, and 1.45-fold, respectively, than that of the WT (*P* < 0.01; Figure 5C). Therefore, OE5 was chosen and used for further transcriptome and metabolome analysis.

### Metabolome profiling of *EbbHLH80*-OE transgenic tobacco

**FIGURE 6:**
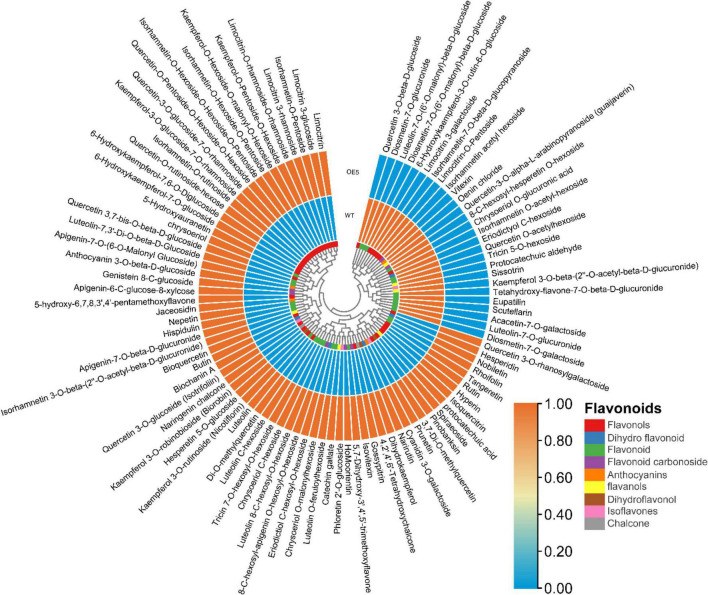
The cluster heat map of flavonoid metabolites between WT and OE5.

To compare metabolite accumulation, leaf samples were analyzed using UPLC−ESI−MS/MS. The metabolite profiles of *EbbHLH80*-OE and WT leaves showed significantly differences. Using the metabolite database, a total of 421 differentially accumulated metabolites were identified. Furthermore, 98 of the metabolites were identified as flavonoid-related, constructing a hierarchical heatmap clustering (Figure 6), including 4 anthocyanins, 2 chalcones, 5 dihydroflavonoids, 3 dihydroflavonols, 3 flavanols, 29 flavonoids, 7 flavonoid carbonosides, 41 flavonols, and 4 isoflavones (Figure 6 and Supplementary Table 6). A clear separation could be observed between *EbbHLH80*-OE and WT, suggesting distinct flavonoid profiles.

Differentially accumulated metabolites (DAMs) were selected using VIP ≥ 1 and *T*-test *P* < 0.05. 214 DAMs were significantly different between the compared samples, and 18 flavonoid metabolites were present at significantly higher levels in the *EbbHLH80*-OE line than in the WT (Supplementary Table 7). Based on the results, the *EbbHLH80* may play a crucial role in regulating flavonoid biosynthesis.

### Identification and functional annotation of differentially expressed genes in *EbbHLH80*-OE and wild-type tobacco leaves

Global gene expression was further profiled in the leaf samples of *EbbHLH80*-OE and WT. High-quality libraries (with Q30 values higher than 91%) were constructed. The libraries contained 46,694,552–63,472,428 clean reads that mapped successfully to the tobacco genome, with matching rates in the range of 94.03–96.50%. Consequently, a total of 9,377 differentially expressed genes (DEGs) were identified by an absolute log2FC > |1| with FDR < 0.05 between *EbbHLH80*-OE and WT, including 4,262 upregulated genes and 5,115 downregulated genes (Supplementary Table 8).

**FIGURE 7:**
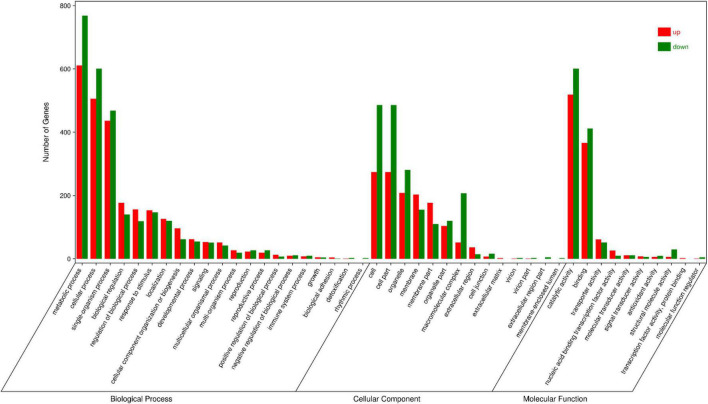
Gene Ontology (GO) analysis of DEGs between WT and OE5.

**FIGURE 8:**
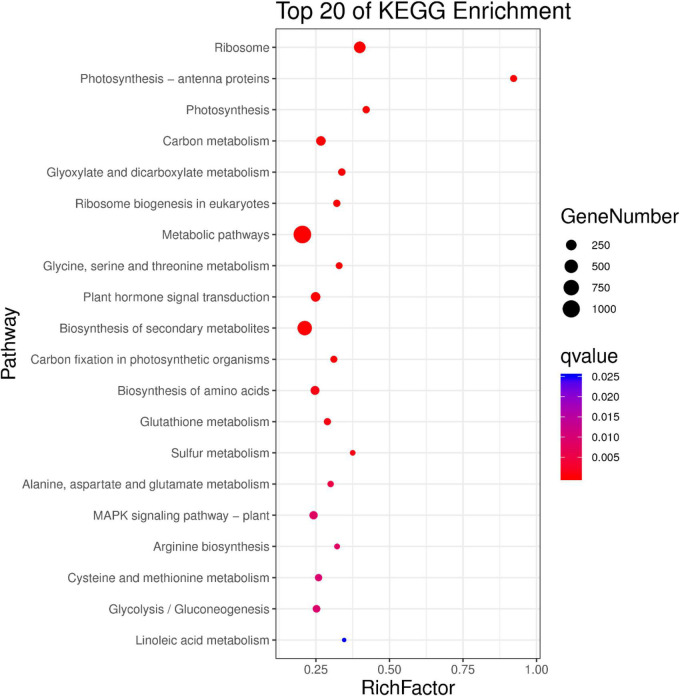
Kyoto Encyclopedia of Genes and Genomes (KEGG) pathway enrichment analysis of DEGs between WT and OE5. The *X*-axis represents the enrichment factor, and the *Y*-axis represents the enrichment gene. The size of the dot represents the number of differentially enriched genes.

GO annotations and KEGG pathway enrichment analysis were used to predict the functions of DEGs. GO annotations assigned these DEGs to 45 functional terms. Twenty-one were categorized into biological processes, 10 into molecular functions, and 14 into cellular components (Figure 7). The genes clustered in the biological processes group were mainly related to metabolic processes (1,379 sequences), cellular processes (1,107 sequences) and single tissue processes (904 sequences). The molecular function terms were related to catalytic activity (660 sequences), binding (778 sequences) and transporter activity (113 sequences). The majority of genes involved in cellular components were located in the cell (760 sequences), cell part (760 sequences) and organelle (561 sequences). Moreover, KEGG pathway enrichment analysis showed that DEGs were significantly enriched in biosynthesis of secondary metabolites, ribosome plant hormone signal transduction and the MAPK signaling pathway (Figure 8 and Supplementary Table 9).

### Expression patterns of differentially expressed genes involved in the flavonoid transport

Predicted coding genes associated with transport were identified by transcriptome data analysis (Supplementary Table 10), including 38 glutathione S–transferase (GST), 24 multidrug and toxic compound extrusion (MATE), and 48 ATP-binding cassette (ABC) transporters. Among them, 20 *ABCs*, 3 *GSTs*, and 7 *MATEs* were significantly upregulated in *EbbHLH80*-OE (FDR < 0.05 and log2FC > |1|), indicating that they may be key flavonoid transport genes.

### Expression patterns of differentially expressed genes involved in transcription factors

Three hundred and fifty-five DEGs were predicted to encode TFs that regulate flavonoid synthesis (Supplementary Table 11). Of these DEGs, there were 78 ethylene response factors (*ERFs*), 22 *MYB*s, 24 *bHLH*s, 45 *WRKY*s, and 3 *NAC*s. Genes annotated as *MYB35* (LOC107815451), *MYB86* (LOC107802100, LOC107762209), *MYB48* (LOC107814331), *MYB32* (LOC107807690), and *MYB44*-like (LOC107816351) were upregulated transcription factors. A significant change in expression was also observed for TFs annotated as *bHLH*, *WRKY*, *NAC*, *MYB*, and *ERF* family members (Supplementary Tables 12–16). In addition, these genes are not only involved in plant growth and development, but also involved in responses to ethylene signaling pathways, biosynthesis of flavonoids and anthocyanins, regulation of the expression of ABA inducible genes, and involvement in resistance to multiple stress responses. Moreover, 1 DEG was identified that was involved in the light signal transduction pathway. Genes associated with GATA transcription factor 9 were annotated as GATA transcription factors (Supplementary Table 17).

### Quantitative real-time PCR validation of the expression patterns of flavonoid-related genes

**FIGURE 9:**
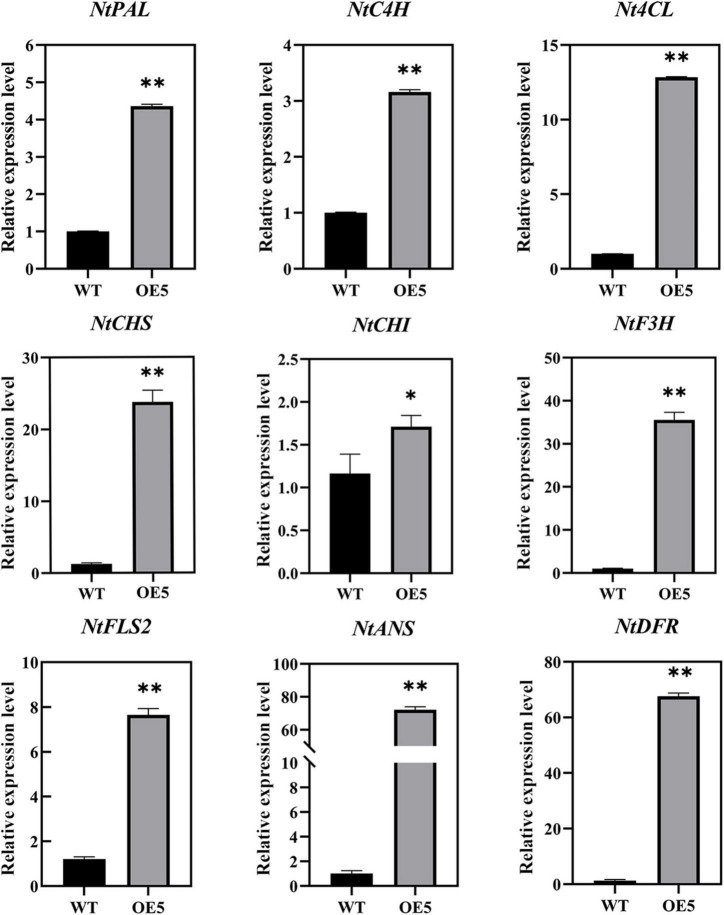
Expression analysis of flavonoid biosynthesis genes between WT and OE5. The characters on the *X*-axis indicate the WT and OE5. The *Y*-axis represents the relative level of gene expression. Data represent mean ± SD of three biological replicates and the one-way analysis of variance was used for statistical analysis (*P < 0.05; **P < 0.01).

An analysis of the expression levels of nine DEGs involved in flavonoid biosynthesis in WT and *EbbHLH80*-OE plants was conducted to further validate the RNA-seq results. The results confirmed that flavonoid biosynthesis and regulatory genes, including *PAL*, *C4H*, *4CL*, *CHS*, *CHI*, *FLS2*, *F3H*, *DFR*, and *ANS*, were upregulated in *EbbHLH80*-OE (Figure 9). A complete correlation was observed between the RT-qPCR and RNA-seq results, which demonstrated the reliability of the RNA-seq data and DEGs analysis.

## Discussion

Since the full genome sequence of *E. breviscapus* was completed (He et al., 2021), we have been able to identify and characterize all members of the *bHLH* family. The *E. breviscapus* genome contains 116 *bHLH genes* whose predominant nuclear location is consistent with their function as transcription factors (Supplementary Table 5). The number of *EbbHLH* genes was smaller than that in Arabidopsis (162) (Bailey et al., 2003), rice (167) (Li et al., 2006), tomato (152) (Wang et al., 2015), cucumber (142) (Li et al., 2020), pepper (122) (Zhang et al., 2020), and pear (197) (Dong et al., 2021). The reason for this may be the difference in genome size between the plants or evolutionary divergence. A previous study showed that the Arabidopsis *bHLH* gene family contained 21 subgroups (Toledo-Ortiz et al., 2003). The phylogenetic analysis showed that the *E. breviscapus bHLH* gene family contained 18 subgroups (Figure 3) with wildly varied gene numbers from 2 (subgroups 2, subgroups 4, and subgroups 21) to 17 (subgroups 18) and three one-gene subgroups 1, subgroups 5, and subgroups 8. Most EbbHLH proteins clustered in the same group share similar motifs, suggesting that these conserved motifs play crucial roles in the group functions. The gene and protein structure analysis showed that the EbbHLH family also has a broad diversity in intron/exon organizations as well the protein motif patterns.

In plants, flavonoids play an important role in pigmentation (Lai et al., 2014). Flavonoids are found in six main groups: anthocyanins, flavan-3-ols, flavanonols, flavanols, flavones, and phenolic acids. Flavonoids are related to plant stress resistance, as they can improve the ability of plants to resist multiple stresses, thereby increasing plant resistance to damage and protecting the plant from disease (Panche et al., 2016). According to recent research, flavonoid structural genes are regulated by *bHLH* transcription factors (Bai et al., 2011). There have been many studies on flavonoid metabolism and accumulation in plants based on transcriptome and targeted metabolome analyses. Analyses of *TT8* inducible overexpression and loss-of-function lines revealed that *TT8* coordinates glycosylation of nucleotides and flavonoids, suggesting that *TT8* controls nucleotide sugar synthesis (Rai et al., 2016). In *TT8* of the *B. napus* cultivar QinYou NO. 7, *TT8* is primarily expressed during seed development. *BnTT8* can produce yellow seed coats, higher fatty acid concentrations, and lower storage protein concentrations in *tt8-4* mutants (Qi et al., 2017). Through the metabolome analysis of *E. breviscapus*, 214 DAMs were identified, 18 of which were flavonoid metabolites. In *EbbHLH80*-OE lines, the contents of flavonoid metabolites are significantly higher compared with WT, and these findings will provide a more comprehensive approach to *EbbHLH80*-mediated regulation of flavonoid biosynthesis.

*EbbHLH80*-OE leaves accumulate significantly more flavonoids than WT, suggesting that this is a direct result of the higher expression of flavonoid biosynthesis genes (*PAL*, *C4H*, *4CL*, *CHI*, *CHS*, *F3H*, *FLS2*, *DFR*, and *ANS*). The correlations between key DEGs and metabolites associated with flavonoid biosynthesis in *EbbHLH80*-OE tobacco leaves were analyzed. Of these, LOC107800382, LOC107781346, LOC107781089, and LOC107824957 showed significant positive correlations with nine types of flavonoid metabolites (Supplementary Figure 1). Previous studies showed that anthocyanin content was positively correlated with the expression of early or late biosynthesis genes (Rahim et al., 2014). The coexpression of flavonoid-related genes in synthesis and metabolism may be a universal regulatory mechanism by which the flavonoid pathway controls structural gene expression. Anthocyanins are transferred from the endoplasmic reticulum to the vacuole by *GSTs* in plants (Mueller et al., 2000). In Arabidopsis, *GSTs* such as *TT19* were found to correlate positively with anthocyanin accumulation (Kitamura et al., 2004). Compared to the WT, we found that the transcription of 3 *GST* genes increased significantly in *EbbHLH80*-OE tobacco leaves, corresponding to higher anthocyanin levels. Thus, upregulated *GSTs* are likely to facilitate anthocyanin transport and increase flavonoid levels within the vacuoles. Additionally, anthocyanin is transported and accumulated by ABC transporters and MATE family members (Goodman et al., 2004; Gomez et al., 2011). In this study, 3 *GSTs*, 7 *MATEs*, and 20 *ABCs* were positively correlated with flavonoid accumulation.

It has been shown that MYB alone, coexpression of MYB and bHLH, or the MYB-bHLH-WD40 complex is sufficient to induce flavonoid accumulation in plants (Xie et al., 2016). Several studies have shown that flavonoid biosynthesis is also controlled by TFs, such as the WRKY and ERF families, as well as by lncRNAs and miRNAs (Yao et al., 2017; Yang et al., 2019; An et al., 2020). According to functional assays, *ERF*s play a role in fruit peel degreening, fruit ripening, and hormone signal transduction (Zhou et al., 2016; Han et al., 2018). Additionally, *ERF*s have recently been discovered to be involved in anthocyanin biosynthesis (An et al., 2020). In this study, 49 *ERF*s were upregulated in *EbbHLH80*-OE compared to WT (Supplementary Table 16). In a previous study, the ethylene response factor (ERF) protein MdERF109 was found to be related to anthocyanin biosynthesis, promoting coloration by directly binding to the promoters of anthocyanin-related genes and simultaneously interacting with anthocyanin biosynthesis genes (Ma et al., 2021).

Taken together, these results suggest that *EbbHLH80* activates *ERF109* TFs, activating genes involved in flavonoid biosynthesis pathways and increasing flavonoid concentrations. This indicated that the highly accumulated flavonoid in *EbbHLH80*-OE might be attributed to the activation of *ERF* genes. However, there will be a need to investigate how the spontaneously changed genes in *EbbHLH80*-OE and evaluate how it could cause the accumulations in the expression levels of these transcription factors and their structural genes.

## Conclusion

The present study provided a comprehensive analysis of the *bHLH* gene family from a genome-wide perspective. In *E. breviscapus*, 116 *EbbHLH* genes are found, divided into 18 subfamilies and distributed unevenly on 9 chromosomes. A phylogenetic analysis of these genes was further supported by their similar exon–intron structures and conserved motif compositions. Combining similar gene expression patterns with integrated metabolomic and transcriptomic analysis, *EbbHLH80*-OE tobacco leaves contain higher flavonoids and have altered gene expression patterns compared with Yunyan87 tobacco leaves. Several key genes involved in flavonoid synthesis were upregulated in the transgenic *EbbHLH80*-OE lines, resulting in significantly higher levels of flavonoid.

## Data availability statement

The original contributions presented in this study are publicly available. This data can be found here: NCBI, SRR19135011–SRR19135016.

## Author contributions

YZ, CZ, and SY conceived and designed the experiments. QG, WS, XL, and CX performed the experiments. GC, GX, XYL, GZ, and XNL analyzed the data. YZ, QG, and CZ wrote the article. All authors contributed to the article and approved the submitted version.

## Footnotes

1
http://Pfam.xfam.org/

2
https://web.expasy.org/protparam/

3
http://www.csbio.sjtu.edu.cn/bioinf/plant-multi/

4
https://www.ncbi.nlm.nih.gov

5
https://meme-suite.org/meme/

6
https://www.arabidopsis.org/
