# Supplementary material for: Genome-wide identification of bHLH transcription factors: Discovery of a candidate regulator related to flavonoid biosynthesis in Erigeron breviscapus
Source: Front Plant Sci. 2022 Sep 14;13:977649. doi: 10.3389/fpls.2022.977649 (PMC9515989; doi:10.3389/fpls.2022.977649)
Supplement: Supplementary file 1 [file Data_Sheet_1.zip › Supplementary data/Supplementary figure.pdf]

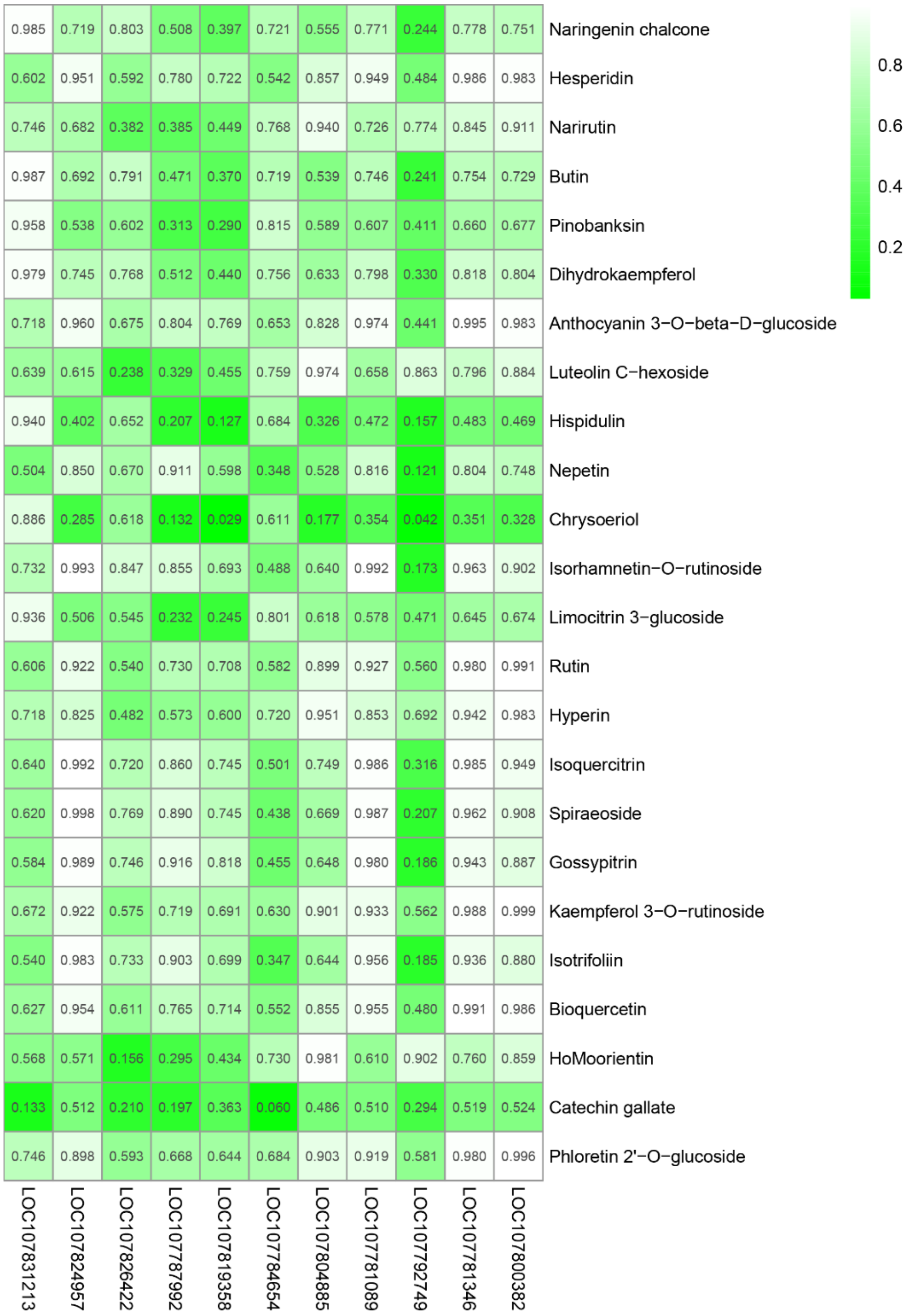

Supplementary Figure 1. Pearson correlation coefficient obtained from gene expression and metabolites involved in flavonoid biosynthesis
