# Supplementary material for: Genome-wide identification of bHLH transcription factors: Discovery of a candidate regulator related to flavonoid biosynthesis in Erigeron breviscapus
Source: Front Plant Sci. 2022 Sep 14;13:977649. doi: 10.3389/fpls.2022.977649 (PMC9515989; doi:10.3389/fpls.2022.977649)
Supplement: Supplementary file 1 [file Data_Sheet_1.zip › Supplementary data/Supplementary table.docx]

**Table S1. Primer sequences used for vector construction**

| **Gene** | **PCR primers** | **size** | **Restriction endonuclease** |
| --- | --- | --- | --- |
| EbbHLH80 | F: ggatcc ATGGCAACCTTTGATTATCCACC | 1896 | BamHI |
|  | R: tctagaTCCTGAAGAGATTATCTCATTAATAG |  | XbaI |

**Table S2. Primer sequence used for gene expression by RT-qPCR**

| **Primer name** | **Sequence** | **Length(bp)** |
| --- | --- | --- |
| EbbHLH80-F  EbbHLH80-R | GCGTTGTCACCTGAGGATCT  ACCGTCTTGGCAAGGATAGC | 20  20 |
| EbACTIN-F  EbACTIN-R | TTGCTGACAGGATGAGCAAG  GAAGCACTTCCTGTGGACAA | 20  20 |
| NtPAL-F  NtPAL-R | TCGCGGTCTTTTCTGAAGTT  ACTGGGGTGATGTTCTGAGG | 20  20 |
| NtC4H-F  NtC4H-R | CACATGAACCTTCACGATGC  GCTCCTCCTACCAACACCAA | 20  20 |
| Nt4CL-F  Nt4CL-R | CCATGAAAGATGAGCAAGCA  GGTACCGTCTCCACGAAAAA | 20  20 |
| NtCHS-F | GGCTCCGTCCATCGGTCAA | 19 |
| NtCHS-R | TCATTGGGTCCACGAAAC | 18 |
| NtCHI-F | TCTCGCCGCTAAATGGAA | 18 |
| NtCHI-R | TACCCGTCAAAGGCAAGA | 18 |
| NtF3H-F | CCATAACATTTGCAGAGATGTAC | 23 |
| NtF3H-R | TCTTCAATGGGCTTGGTTTTCA | 22 |
| NtFLS2-F | AAAACTCCAGGGTCTCAG | 18 |
| NtFLS2-R | CTGTAGGAGGGAGGATTT | 18 |
| NtDFR-F | CTAGCTTAATCACTGCCCTTTCA | 23 |
| NtDFR-R | TAGTACTCCGGCCATTTCTCTT | 22 |
| NtANS-F | GCTATCCCTAAAGAGTATGT | 20 |
| NtANS -R | TCTTTGTGGCATTTCTCG | 18 |
| NtACTIN-F  NtACTIN-R | CTGAGGTCCTTTTCCAACCA  TACCCGGGAACATGGTAGAG | 20  20 |

**Table S3. Renaming of the *EbbHLH*s gene in** ***Erigeron breviscapus***

| **Gene ID** | **Rename** | **Gene ID** | **Rename** |
| --- | --- | --- | --- |
| evm.model.ctg1403.10 | *EbbHLH001* | evm.model.ctg4740.3 | *EbbHLH044* |
| evm.model.ctg5561.3 | *EbbHLH002* | evm.model.ctg4740.1 | *EbbHLH045* |
| evm.model.ctg310.7 | *EbbHLH003* | evm.model.ctg3042.24 | *EbbHLH046* |
| evm.model.ctg5499.5 | *EbbHLH004* | evm.model.ctg544.6 | *EbbHLH047* |
| evm.model.ctg4154.10 | *EbbHLH005* | evm.model.ctg8741.3 | *EbbHLH048* |
| evm.model.ctg9908.1 | *EbbHLH006* | evm.model.ctg86.4 | *EbbHLH049* |
| evm.model.ctg11919.2 | *EbbHLH007* | evm.model.ctg199.4 | *EbbHLH050* |
| evm.model.ctg672.3 | *EbbHLH008* | evm.model.ctg199.1 | *EbbHLH051* |
| evm.model.ctg391.3 | *EbbHLH009* | evm.model.ctg295.5 | *EbbHLH052* |
| evm.model.ctg1960.4 | *EbbHLH010* | evm.model.ctg138.18 | *EbbHLH053* |
| evm.model.ctg9613.5 | *EbbHLH011* | evm.model.ctg4851.3 | *EbbHLH054* |
| evm.model.ctg1518.2 | *EbbHLH012* | evm.model.ctg9475.1 | *EbbHLH055* |
| evm.model.ctg1614.3 | *EbbHLH013* | evm.model.ctg5341.4 | *EbbHLH056* |
| evm.model.ctg104.15 | *EbbHLH014* | evm.model.ctg601.3 | *EbbHLH057* |
| evm.model.ctg104.17 | *EbbHLH015* | evm.model.ctg1589.4 | *EbbHLH058* |
| evm.model.ctg281.22 | *EbbHLH016* | evm.model.ctg687.2 | *EbbHLH059* |
| evm.model.ctg2840.9 | *EbbHLH017* | evm.model.ctg194.9 | *EbbHLH060* |
| evm.model.ctg26.22 | *EbbHLH018* | evm.model.ctg430.3 | *EbbHLH061* |
| evm.model.ctg3.13 | *EbbHLH019* | evm.model.ctg288.11 | *EbbHLH062* |
| evm.model.ctg4630.2 | *EbbHLH020* | evm.model.ctg5725.5 | *EbbHLH063* |
| evm.model.ctg1082.3 | *EbbHLH021* | evm.model.ctg1276.6 | *EbbHLH064* |
| evm.model.ctg6615.3 | *EbbHLH022* | evm.model.ctg1221.7 | *EbbHLH065* |
| evm.model.ctg2462.1 | *EbbHLH023* | evm.model.ctg6514.3 | *EbbHLH066* |
| evm.model.ctg2128.1 | *EbbHLH024* | evm.model.ctg1971.10 | *EbbHLH067* |
| evm.model.ctg298.15 | *EbbHLH025* | evm.model.ctg282.10 | *EbbHLH068* |
| evm.model.ctg136.3 | *EbbHLH026* | evm.model.ctg506.9 | *EbbHLH069* |
| evm.model.ctg2610.5 | *EbbHLH027* | evm.model.ctg3270.5 | *EbbHLH070* |
| evm.model.ctg1014.14 | *EbbHLH028* | evm.model.ctg2789.2 | *EbbHLH071* |
| evm.model.ctg632.20 | *EbbHLH029* | evm.model.ctg2664.1 | *EbbHLH072* |
| evm.model.ctg4551.4 | *EbbHLH030* | evm.model.ctg14778.2 | *EbbHLH073* |
| evm.model.ctg398.8 | *EbbHLH031* | evm.model.ctg5093.5 | *EbbHLH074* |
| evm.model.ctg750.14 | *EbbHLH032* | evm.model.ctg1711.1 | *EbbHLH075* |
| evm.model.ctg178.3 | *EbbHLH033* | evm.model.ctg2386.5 | *EbbHLH076* |
| evm.model.ctg9646.3 | *EbbHLH034* | evm.model.ctg512.4 | *EbbHLH077* |
| evm.model.ctg1932.4 | *EbbHLH035* | evm.model.ctg4976.6 | *EbbHLH078* |
| evm.model.ctg119.15 | *EbbHLH036* | evm.model.ctg9683.3 | *EbbHLH079* |
| evm.model.ctg16262.1 | *EbbHLH037* | evm.model.ctg4334.5 | *EbbHLH080* |
| evm.model.ctg4971.1 | *EbbHLH038* | evm.model.ctg1624.5 | *EbbHLH081* |
| evm.model.ctg2259.3 | *EbbHLH039* | evm.model.ctg2244.6 | *EbbHLH082* |
| evm.model.ctg2495.2 | *EbbHLH040* | evm.model.ctg2598.4 | *EbbHLH083* |
| evm.model.ctg1169.11 | *EbbHLH041* | evm.model.ctg1822.6 | *EbbHLH084* |
| evm.model.ctg6782.5 | *EbbHLH042* | evm.model.ctg221.8 | *EbbHLH085* |
| evm.model.ctg1402.7 | *EbbHLH043* | evm.model.ctg12747.2 | *EbbHLH086* |
| evm.model.ctg1503.4 | *EbbHLH087* | evm.model.ctg3305.6 | *EbbHLH102* |
| evm.model.ctg1869.5 | *EbbHLH088* | evm.model.ctg6081.6 | *EbbHLH103* |
| evm.model.ctg1065.4 | *EbbHLH089* | evm.model.ctg2447.3 | *EbbHLH104* |
| evm.model.ctg2012.11 | *EbbHLH090* | evm.model.ctg3488.3 | *EbbHLH105* |
| evm.model.ctg1204.3 | *EbbHLH091* | evm.model.ctg45.4 | *EbbHLH106* |
| evm.model.ctg1895.8 | *EbbHLH092* | evm.model.ctg134.18 | *EbbHLH107* |
| evm.model.ctg338.10 | *EbbHLH093* | evm.model.ctg10877.3 | *EbbHLH108* |
| evm.model.ctg625.24 | *EbbHLH094* | evm.model.ctg5329.1 | *EbbHLH109* |
| evm.model.ctg154.2 | *EbbHLH095* | evm.model.ctg1011.1 | *EbbHLH110* |
| evm.model.ctg58.5 | *EbbHLH096* | evm.model.ctg2955.11 | *EbbHLH111* |
| evm.model.ctg20.4 | *EbbHLH097* | evm.model.ctg4833.2 | *EbbHLH112* |
| evm.model.ctg12014.1 | *EbbHLH098* | evm.model.ctg517.5 | *EbbHLH113* |
| evm.model.ctg9865.3 | *EbbHLH099* | evm.model.ctg2276.5 | *EbbHLH114* |
| evm.model.ctg909.3 | *EbbHLH100* | evm.model.ctg50.17 | *EbbHLH115* |
| evm.model.ctg3305.5 | *EbbHLH101* | evm.model.ctg4273.4 | *EbbHLH116* |

**Table S4. Physicochemical properties prediction of EbbHLHs in *Erigeron Breviscapus***

|  | **Physical position** | | | **Property of EbbHLH protein** | | |
| --- | --- | --- | --- | --- | --- | --- |
| **Gene** | **Chr.NO** | **Start position**  **/bp** | **End position**  **/bp** | **Length**  **/aa** | **pI** | **Molecular weight**  **/D** |
| EbbHLH001 | Chr1 | 122575 | 124283 | 244 | 6.01 | 27209.58 |
| EbbHLH002 | Chr1 | 32449 | 36040 | 327 | 5.86 | 36006.97 |
| EbbHLH003 | Chr1 | 116859 | 120130 | 558 | 5.25 | 63972.17 |
| EbbHLH004 | Chr1 | 33669 | 35601 | 239 | 7.06 | 26457.96 |
| EbbHLH005 | Chr1 | 161049 | 167547 | 328 | 6.1 | 36341.02 |
| EbbHLH006 | Chr1 | 32860 | 35647 | 254 | 5.83 | 28454.63 |
| EbbHLH007 | Chr1 | 8516 | 10017 | 314 | 7.29 | 34967.34 |
| EbbHLH008 | Chr1 | 32861 | 35857 | 431 | 5.74 | 48167.92 |
| EbbHLH009 | Chr1 | 67593 | 71624 | 412 | 8.17 | 46023.15 |
| EbbHLH010 | Chr1 | 182006 | 184111 | 332 | 6.39 | 36702.16 |
| EbbHLH011 | Chr1 | 33671 | 35915 | 441 | 6.43 | 48710.62 |
| EbbHLH012 | Chr1 | 83985 | 85482 | 314 | 7.29 | 34967.34 |
| EbbHLH013 | Chr1 | 47556 | 52500 | 491 | 8.22 | 54462.38 |
| EbbHLH014 | Chr1 | 193358 | 194131 | 106 | 10.11 | 12092.03 |
| EbbHLH015 | Chr1 | 259347 | 261797 | 495 | 5.86 | 53546.67 |
| EbbHLH016 | Chr1 | 431326 | 434493 | 406 | 5.97 | 45375.58 |
| EbbHLH017 | Chr1 | 236636 | 237311 | 95 | 6.72 | 10806.05 |
| EbbHLH018 | Chr1 | 406642 | 409865 | 364 | 5.58 | 41383.77 |
| EbbHLH019 | Chr1 | 218379 | 219667 | 324 | 8.44 | 35411.43 |
| EbbHLH020 | Chr1 | 33530 | 39093 | 317 | 7.75 | 35148.29 |
| EbbHLH021 | Chr1 | 71762 | 74162 | 400 | 5.96 | 45201.93 |
| EbbHLH022 | Chr1 | 41304 | 43349 | 404 | 7.92 | 45640.32 |
| EbbHLH023 | Chr1 | 19350 | 21598 | 267 | 8.82 | 29472.44 |
| EbbHLH024 | Chr1 | 6982 | 12882 | 300 | 5.63 | 32980.87 |
| EbbHLH025 | Chr1 | 327507 | 329369 | 226 | 9.14 | 25170.3 |
| EbbHLH026 | Chr1 | 36055 | 38227 | 518 | 6.13 | 57349.63 |
| EbbHLH027 | Chr1 | 92783 | 95140 | 500 | 5.39 | 53498.02 |
| EbbHLH028 | Chr1 | 276904 | 279982 | 361 | 5.84 | 40233.43 |
| EbbHLH029 | Chr2 | 214071 | 217963 | 243 | 6.22 | 26994.16 |
| EbbHLH030 | Chr2 | 35832 | 40267 | 824 | 6.08 | 93563.26 |
| EbbHLH031 | Chr2 | 142607 | 145082 | 269 | 8.85 | 30159.29 |
| EbbHLH032 | Chr2 | 126642 | 128939 | 251 | 7.71 | 28063.98 |
| EbbHLH033 | Chr2 | 43938 | 52600 | 542 | 9.39 | 61556.25 |
| EbbHLH034 | Chr2 | 35668 | 38320 | 342 | 6.07 | 38536.86 |
| EbbHLH035 | Chr2 | 81394 | 82538 | 247 | 6.5 | 28012.68 |
| EbbHLH036 | Chr2 | 258138 | 259193 | 261 | 5.84 | 28964.45 |
| EbbHLH037 | Chr2 | 3714 | 5410 | 414 | 4.97 | 47111.68 |
| EbbHLH038 | Chr2 | 14621 | 16093 | 333 | 5.31 | 37661.75 |
| EbbHLH039 | Chr2 | 76217 | 77872 | 333 | 4.8 | 37935.49 |
| EbbHLH040 | Chr2 | 22092 | 28410 | 251 | 6.11 | 28314.52 |
| EbbHLH041 | Chr2 | 117819 | 120984 | 269 | 5.53 | 28885.96 |
| EbbHLH042 | Chr2 | 62098 | 64346 | 267 | 8.82 | 29472.44 |
| EbbHLH043 | Chr2 | 110698 | 112569 | 339 | 6.76 | 38308.66 |
| EbbHLH044 | Chr3 | 134243 | 135938 | 325 | 5.35 | 36606.54 |
| EbbHLH045 | Chr3 | 61945 | 63602 | 344 | 6.16 | 38565.33 |
| EbbHLH046 | Chr3 | 311118 | 313123 | 257 | 7.02 | 29070.96 |
| EbbHLH047 | Chr3 | 103615 | 104719 | 235 | 9.06 | 26625.95 |
| EbbHLH048 | Chr3 | 34087 | 37266 | 288 | 8.59 | 30834.68 |
| EbbHLH049 | Chr3 | 57364 | 59060 | 414 | 4.97 | 47111.68 |
| EbbHLH050 | Chr3 | 83134 | 83734 | 89 | 8.93 | 10061.31 |
| EbbHLH051 | Chr3 | 35565 | 36604 | 93 | 9.09 | 10682.91 |
| EbbHLH052 | Chr3 | 116071 | 118279 | 261 | 8.15 | 29485.48 |
| EbbHLH053 | Chr3 | 206701 | 209547 | 286 | 5.4 | 30997.09 |
| EbbHLH054 | Chr4 | 20572 | 22958 | 383 | 5.71 | 43229.01 |
| EbbHLH055 | Chr4 | 3405 | 8004 | 340 | 5.09 | 38142.82 |
| EbbHLH056 | Chr4 | 63223 | 67403 | 316 | 6.33 | 35360.29 |
| EbbHLH057 | Chr4 | 113361 | 114512 | 305 | 6.71 | 34087.96 |
| EbbHLH058 | Chr4 | 40638 | 42568 | 390 | 5.96 | 43976.78 |
| EbbHLH059 | Chr4 | 150682 | 153889 | 380 | 6.18 | 42840.78 |
| EbbHLH060 | Chr4 | 186533 | 189161 | 275 | 6.15 | 31058.98 |
| EbbHLH061 | Chr4 | 9152 | 10477 | 287 | 5.27 | 32259.93 |
| EbbHLH062 | Chr4 | 438988 | 440575 | 223 | 5.6 | 25762.04 |
| EbbHLH063 | Chr4 | 45707 | 47341 | 168 | 8.97 | 18738.98 |
| EbbHLH064 | Chr4 | 127546 | 135671 | 584 | 6.43 | 63968.88 |
| EbbHLH065 | Chr5 | 73739 | 77352 | 286 | 5.38 | 31549.2 |
| EbbHLH066 | Chr5 | 18892 | 31493 | 510 | 6.6 | 55140.31 |
| EbbHLH067 | Chr5 | 282117 | 283468 | 235 | 8.29 | 26712.66 |
| EbbHLH068 | Chr5 | 124169 | 128662 | 403 | 8.5 | 45466.2 |
| EbbHLH069 | Chr5 | 212429 | 214483 | 504 | 6.27 | 55416.16 |
| EbbHLH070 | Chr5 | 102347 | 104310 | 443 | 7.03 | 49291.41 |
| EbbHLH071 | Chr5 | 88683 | 90438 | 257 | 8.87 | 28957.1 |
| EbbHLH072 | Chr5 | 10630 | 11913 | 272 | 6.13 | 30883.67 |
| EbbHLH073 | Chr5 | 11162 | 13851 | 240 | 9.38 | 26077.02 |
| EbbHLH074 | Chr5 | 49918 | 52460 | 323 | 5.69 | 36349.27 |
| EbbHLH075 | Chr5 | 43938 | 52600 | 631 | 5.19 | 70862.05 |
| EbbHLH076 | Chr5 | 133497 | 137282 | 295 | 7.14 | 33312.61 |
| EbbHLH077 | Chr5 | 116664 | 121407 | 297 | 5.68 | 33674.23 |
| EbbHLH078 | Chr6 | 115243 | 119501 | 388 | 5.97 | 43452.85 |
| EbbHLH079 | Chr6 | 25714 | 27136 | 337 | 6.68 | 38063.93 |
| EbbHLH080 | Chr6 | 127047 | 131320 | 631 | 5.19 | 70862.05 |
| EbbHLH081 | Chr6 | 116061 | 117253 | 280 | 5.06 | 31218.72 |
| EbbHLH082 | Chr6 | 42598 | 44347 | 331 | 6.72 | 37391.53 |
| EbbHLH083 | Chr6 | 92837 | 95104 | 190 | 6.44 | 21346.52 |
| EbbHLH084 | Chr6 | 93624 | 97595 | 183 | 5.3 | 21131.84 |
| EbbHLH085 | Chr6 | 237288 | 239399 | 194 | 9.33 | 21699.3 |
| EbbHLH086 | Chr6 | 18062 | 20174 | 194 | 9.33 | 21699.3 |
| EbbHLH087 | Chr7 | 11640 | 13192 | 353 | 5.09 | 39685.73 |
| EbbHLH088 | Chr7 | 79763 | 81567 | 336 | 5.62 | 37521.29 |
| EbbHLH089 | Chr7 | 95587 | 100696 | 570 | 6.69 | 61916.76 |
| EbbHLH090 | Chr7 | 164257 | 170159 | 534 | 6.82 | 58622.38 |
| EbbHLH091 | Chr7 | 119250 | 121710 | 279 | 8.21 | 30647.3 |
| EbbHLH092 | Chr7 | 169330 | 172908 | 625 | 5.27 | 71596.45 |
| EbbHLH093 | Chr7 | 178351 | 180043 | 137 | 8.83 | 15879.55 |
| EbbHLH094 | Chr7 | 289395 | 291952 | 262 | 9.92 | 28706.03 |
| EbbHLH095 | Chr7 | 24996 | 28425 | 311 | 5.93 | 33379.27 |
| EbbHLH096 | Chr7 | 64968 | 67882 | 299 | 8.53 | 32838.69 |
| EbbHLH097 | Chr7 | 19937 | 21618 | 183 | 7.7 | 20449.86 |
| EbbHLH098 | Chr7 | 3515 | 4190 | 95 | 6.72 | 10806.05 |
| EbbHLH099 | Chr7 | 33767 | 34442 | 95 | 6.72 | 10806.05 |
| EbbHLH100 | Chr8 | 50187 | 51930 | 386 | 5.91 | 43118.84 |
| EbbHLH101 | Chr8 | 83786 | 87709 | 316 | 6.27 | 35370.76 |
| EbbHLH102 | Chr8 | 89439 | 92328 | 173 | 9.62 | 19904.64 |
| EbbHLH103 | Chr8 | 53898 | 57532 | 194 | 9.2 | 22325.75 |
| EbbHLH104 | Chr8 | 47279 | 51110 | 470 | 8.77 | 51041.62 |
| EbbHLH105 | Chr8 | 67579 | 71966 | 515 | 6.79 | 58199.8 |
| EbbHLH106 | Chr8 | 56550 | 57527 | 165 | 8.99 | 18464.46 |
| EbbHLH107 | Chr8 | 296832 | 302287 | 397 | 5.6 | 41998.6 |
| EbbHLH108 | Chr8 | 20219 | 26505 | 534 | 6.82 | 58622.38 |
| EbbHLH091 | Chr7 | 119250 | 121710 | 279 | 8.21 | 30647.3 |
| EbbHLH092 | Chr7 | 169330 | 172908 | 625 | 5.27 | 71596.45 |
| EbbHLH093 | Chr7 | 178351 | 180043 | 137 | 8.83 | 15879.55 |
| EbbHLH094 | Chr7 | 289395 | 291952 | 262 | 9.92 | 28706.03 |
| EbbHLH095 | Chr7 | 24996 | 28425 | 311 | 5.93 | 33379.27 |
| EbbHLH096 | Chr7 | 64968 | 67882 | 299 | 8.53 | 32838.69 |
| EbbHLH097 | Chr7 | 19937 | 21618 | 183 | 7.7 | 20449.86 |
| EbbHLH098 | Chr7 | 3515 | 4190 | 95 | 6.72 | 10806.05 |
| EbbHLH099 | Chr7 | 33767 | 34442 | 95 | 6.72 | 10806.05 |
| EbbHLH100 | Chr8 | 50187 | 51930 | 386 | 5.91 | 43118.84 |
| EbbHLH101 | Chr8 | 83786 | 87709 | 316 | 6.27 | 35370.76 |
| EbbHLH102 | Chr8 | 89439 | 92328 | 173 | 9.62 | 19904.64 |
| EbbHLH103 | Chr8 | 53898 | 57532 | 194 | 9.2 | 22325.75 |
| EbbHLH104 | Chr8 | 47279 | 51110 | 470 | 8.77 | 51041.62 |
| EbbHLH105 | Chr8 | 67579 | 71966 | 515 | 6.79 | 58199.8 |
| EbbHLH106 | Chr8 | 56550 | 57527 | 165 | 8.99 | 18464.46 |
| EbbHLH107 | Chr8 | 296832 | 302287 | 397 | 5.6 | 41998.6 |
| EbbHLH108 | Chr8 | 20219 | 26505 | 534 | 6.82 | 58622.38 |
| EbbHLH109 | Chr9 | 14629 | 15903 | 315 | 5.09 | 36320.86 |
| EbbHLH110 | Chr9 | 1215 | 5628 | 756 | 5.51 | 84600.9 |
| EbbHLH111 | Chr9 | 251704 | 255585 | 321 | 5.05 | 36345.7 |
| EbbHLH112 | Chr9 | 16792 | 18209 | 225 | 9.07 | 25626.98 |
| EbbHLH113 | Chr9 | 107154 | 110337 | 870 | 9.4 | 100097.86 |
| EbbHLH114 | Chr9 | 45042 | 47868 | 368 | 4.96 | 41319.82 |
| EbbHLH115 | Chr9 | 267172 | 269120 | 344 | 7.3 | 37341.21 |
| EbbHLH116 | Chr9 | 102287 | 103837 | 311 | 6.37 | 34422.94 |

**Table S5. The subcellular localization of EbbHLHs**

| **Gene Name** | **Subcellular Localization** | **Gene Name** | **Subcellular Localization** | **Gene Name** | **Subcellular Localization** |
| --- | --- | --- | --- | --- | --- |
| EbbHLH001 | Nucleus. | EbbHLH040 | Nucleus. | EbbHLH079 | Nucleus. |
| EbbHLH002 | Nucleus. | EbbHLH041 | Nucleus. | EbbHLH080 | Nucleus. |
| EbbHLH003 | Nucleus. | EbbHLH042 | Nucleus. | EbbHLH081 | Nucleus. |
| EbbHLH004 | Nucleus. | EbbHLH043 | Nucleus. | EbbHLH082 | Nucleus. |
| EbbHLH005 | Nucleus. | EbbHLH044 | Nucleus. | EbbHLH083 | Nucleus. |
| EbbHLH006 | Nucleus. | EbbHLH045 | Nucleus. | EbbHLH084 | Nucleus. |
| EbbHLH007 | Nucleus. | EbbHLH046 | Nucleus. | EbbHLH085 | Nucleus. |
| EbbHLH008 | Nucleus. | EbbHLH047 | Nucleus. | EbbHLH086 | Nucleus. |
| EbbHLH009 | Nucleus. | EbbHLH048 | Nucleus. | EbbHLH087 | Nucleus. |
| EbbHLH010 | Nucleus. | EbbHLH049 | Nucleus. | EbbHLH088 | Nucleus. |
| EbbHLH011 | Nucleus. | EbbHLH050 | Nucleus. | EbbHLH089 | Nucleus. |
| EbbHLH012 | Nucleus. | EbbHLH051 | Nucleus. | EbbHLH090 | Nucleus. |
| EbbHLH013 | Nucleus. | EbbHLH052 | Nucleus. | EbbHLH091 | Nucleus. |
| EbbHLH014 | Nucleus. | EbbHLH053 | Nucleus. | EbbHLH092 | Nucleus. |
| EbbHLH015 | Nucleus. | EbbHLH054 | Nucleus. | EbbHLH093 | Nucleus. |
| EbbHLH016 | Nucleus. | EbbHLH055 | Nucleus. | EbbHLH094 | Mitochondrion. |
| EbbHLH017 | Nucleus. | EbbHLH056 | Nucleus. | EbbHLH095 | Nucleus. |
| EbbHLH018 | Nucleus. | EbbHLH057 | Nucleus. | EbbHLH096 | Nucleus. |
| EbbHLH019 | Nucleus. | EbbHLH058 | Nucleus. | EbbHLH097 | Nucleus. |
| EbbHLH020 | Nucleus. | EbbHLH059 | Nucleus. | EbbHLH098 | Nucleus. |
| EbbHLH021 | Nucleus. | EbbHLH060 | Nucleus. | EbbHLH099 | Nucleus. |
| EbbHLH022 | Nucleus. | EbbHLH061 | Nucleus. | EbbHLH100 | Nucleus. |
| EbbHLH023 | Nucleus. | EbbHLH062 | Nucleus. | EbbHLH101 | Nucleus. |
| EbbHLH024 | Nucleus. | EbbHLH063 | Nucleus. | EbbHLH102 | Nucleus. |
| EbbHLH025 | Nucleus. | EbbHLH064 | Nucleus. | EbbHLH103 | Nucleus. |
| EbbHLH026 | Nucleus. | EbbHLH065 | Nucleus. | EbbHLH104 | Nucleus. |
| EbbHLH027 | Nucleus. | EbbHLH066 | Nucleus. | EbbHLH105 | Nucleus. |
| EbbHLH028 | Nucleus. | EbbHLH067 | Nucleus. | EbbHLH106 | Nucleus. |
| EbbHLH029 | Nucleus. | EbbHLH068 | Nucleus. | EbbHLH107 | Nucleus. |
| EbbHLH030 | Nucleus. | EbbHLH069 | Nucleus. | EbbHLH108 | Nucleus. |
| EbbHLH031 | Nucleus. | EbbHLH070 | Nucleus. | EbbHLH109 | Nucleus. |
| EbbHLH032 | Nucleus. | EbbHLH071 | Nucleus. | EbbHLH110 | Nucleus. |
| EbbHLH033 | Nucleus. | EbbHLH072 | Nucleus. | EbbHLH111 | Nucleus. |
| EbbHLH034 | Nucleus. | EbbHLH073 | Nucleus. | EbbHLH112 | Nucleus. |
| EbbHLH035 | Nucleus. | EbbHLH074 | Nucleus. | EbbHLH113 | Nucleus. |
| EbbHLH036 | Nucleus. | EbbHLH075 | Nucleus. | EbbHLH114 | Nucleus. |
| EbbHLH037 | Nucleus. | EbbHLH076 | Nucleus. | EbbHLH115 | Nucleus. |
| EbbHLH038 | Nucleus. | EbbHLH077 | Nucleus. | EbbHLH116 | Nucleus. |
| EbbHLH039 | Nucleus. | EbbHLH078 | Nucleus. |  |  |
